# Management of Multiple Adjacent Gingival Recessions Using the Zucchelli Modification of the Coronally Advanced Flap Without Connective Tissue Grafting: A Case Report

**DOI:** 10.7759/cureus.114889

**Published:** 2026-08-20

**Authors:** Keily J Portillo Avila, Carlos A Orozco Hernandez, Maray Folgoso, Diosbeny N Camacho Grullon, Daniela Fernandez Hlebajna

**Affiliations:** 1 Periodontics, Dental Loft, San Pedro Sula, HND; 2 Dentistry, Fortune Dental, Kissimmee, USA; 3 Dentistry, Real Smile Dentistry, Miami, USA; 4 Dentistry, Expert Dental NYC, New York, USA; 5 Dentistry, Lakeside Family Dentistry, Apopka, USA

**Keywords:** coronally advanced flap, esthetic outcomes, gingival recession, hiv infection, mucogingival defects, multiple recessions, periodontal plastic surgery, periodontal surgery, root coverage, zucchelli technique

## Abstract

Gingival recession is a common periodontal condition characterized by apical migration of the gingival margin, resulting in root surface exposure, dentinal hypersensitivity, esthetic concerns, and increased susceptibility to root caries. Multiple adjacent gingival recessions present a therapeutic challenge because treatment aims to achieve root coverage while preserving gingival harmony and esthetic outcomes. The Zucchelli modification of the coronally advanced flap (CAF) has been widely used for the management of multiple recession defects due to its ability to achieve coronal advancement without vertical releasing incisions while preserving the interdental papillae.

This case report describes the treatment of multiple adjacent maxillary gingival recessions in a 45-year-old female with well-controlled HIV infection receiving antiretroviral therapy. The patient presented with root sensitivity and esthetic concerns associated with gingival recession affecting the maxillary anterior region. Following comprehensive clinical and periodontal evaluation, root coverage therapy was performed using the Zucchelli modification of the CAF without connective tissue grafting. After meticulous root surface preparation and tension-free coronal advancement, the flap was stabilized with resorbable sutures.

Postoperative healing was uneventful, and follow-up examination demonstrated increased root coverage, improved gingival contour, reduction of root exposure, and enhanced esthetics. The patient reported significant improvement in both function and appearance. In this case, the Zucchelli modification of the CAF without connective tissue grafting was feasible in a carefully selected patient with medically controlled HIV infection and was associated with favorable clinical healing and esthetic improvement during follow-up.

## Introduction

Gingival recession is characterized by the apical migration of the gingival margin beyond the cementoenamel junction, resulting in exposure of the root surface. This condition may lead to dentinal hypersensitivity, root caries, plaque accumulation, cervical abrasion, and esthetic concerns, particularly when affecting the maxillary anterior dentition. The etiology of gingival recession is multifactorial and includes traumatic toothbrushing, periodontal inflammation, thin periodontal phenotype, tooth malposition, occlusal trauma, and other local or systemic factors that may influence periodontal health and wound healing [1,2].

Periodontal plastic surgery has evolved considerably over the past decades and has become a predictable treatment modality for the management of gingival recession defects [3,4]. Several surgical procedures have been developed to achieve root coverage and improve soft tissue esthetics. Among these, the coronally advanced flap (CAF) is considered one of the most predictable approaches for the treatment of gingival recession defects [5]. The modification described by Zucchelli for the treatment of multiple adjacent gingival recessions improved flap design, vascularization, and esthetic outcomes while avoiding vertical releasing incisions [6]. Subsequent systematic reviews and clinical studies have further supported the effectiveness of modified CAF procedures for root coverage and soft tissue augmentation [7-12].

Recent evidence has also highlighted the importance of recognizing complications, technical considerations, and long-term outcomes associated with root coverage procedures [13,14]. Human immunodeficiency virus (HIV) infection is a chronic medical condition that may influence oral health and periodontal status. Oral manifestations remain common in affected individuals, although advances in antiretroviral therapy have significantly improved immune function, life expectancy, and overall quality of life [15]. Historical and contemporary evidence supports the use of periodontal plastic surgery to improve both function and esthetics in appropriately selected patients [16-18].

Current evidence further emphasizes the importance of gingival phenotype and soft tissue thickness in determining the long-term stability and success of root coverage procedures [19,20]. In the present case, the Zucchelli modification of the CAF was performed without connective tissue grafting to treat multiple adjacent recession defects while avoiding the additional surgical morbidity associated with harvesting tissue from a second donor site. This consideration was particularly relevant in a patient with medically controlled HIV infection, in whom systemic health and wound healing were carefully considered during treatment planning. Although CAF procedures are well established for the treatment of gingival recession, clinical reports describing this graft-free approach in medically controlled HIV-positive patients remain limited.

This case report describes the management of multiple adjacent maxillary gingival recessions in a medically controlled HIV positive patient using the Zucchelli modification of the CAF without connective tissue grafting. The clinical relevance of this case lies in documenting the feasibility and healing response of this less invasive approach in a carefully selected patient while avoiding a second surgical donor site.

## Case presentation

A 45-year-old female presented to the dental clinic with complaints of root sensitivity and dissatisfaction with the appearance of her gingiva in the maxillary left anterior region. Her medical history was significant for HIV infection that was well controlled with antiretroviral therapy (Biktarvy, one tablet daily). The patient reported compliance with her medication regimen and denied any history of opportunistic infections. She reported no known drug allergies and was classified as American Society of Anesthesiologists (ASA) Physical Status II. Prior to surgical intervention, the patient’s medical history and current antiretroviral therapy were reviewed, confirming adherence to treatment and medically controlled HIV infection. Standard infection control precautions and routine surgical aseptic protocols were followed throughout treatment.

**Figure 1:**
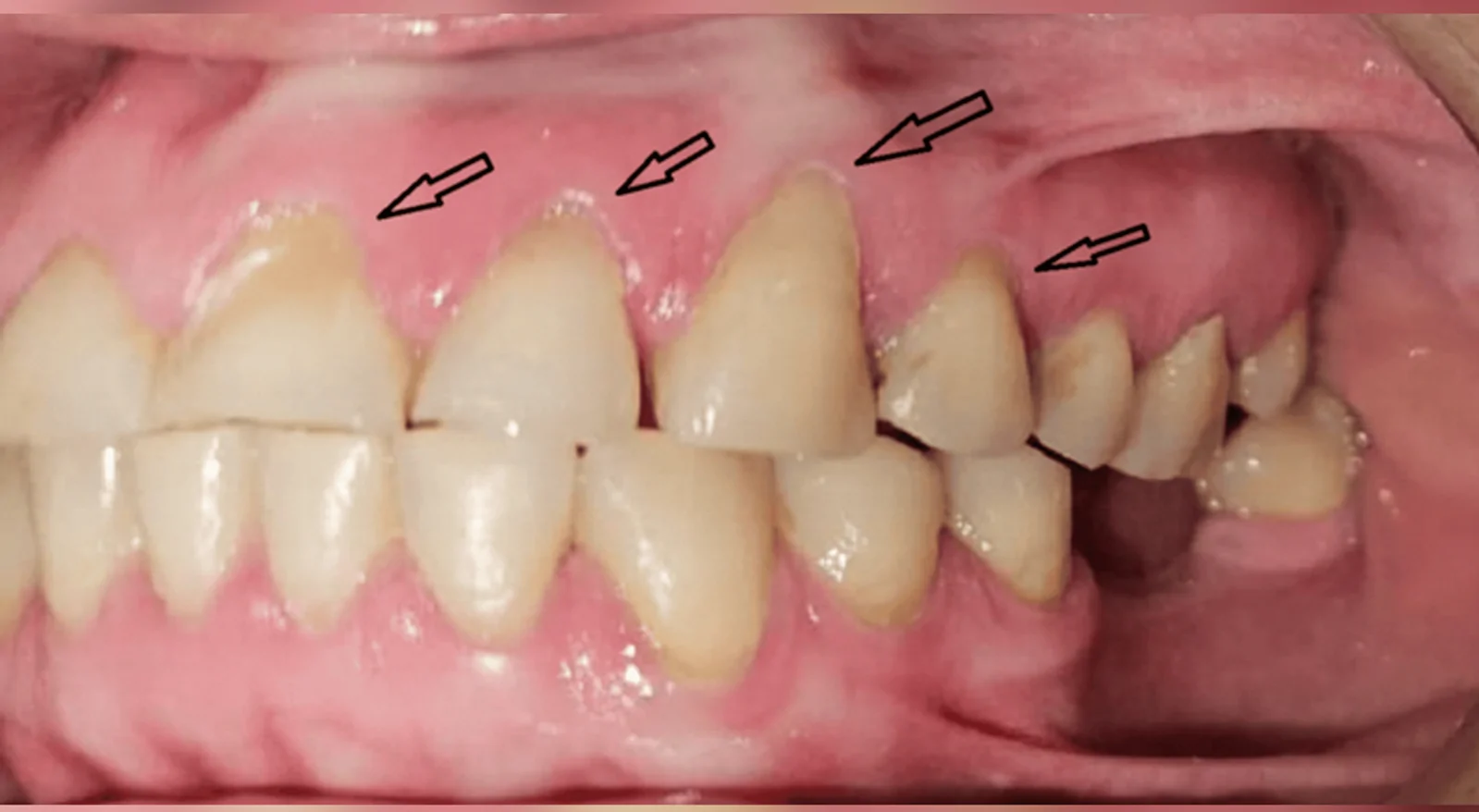
Preoperative Clinical View of Multiple Adjacent Gingival Recessions. Preoperative intraoral photograph demonstrating multiple adjacent gingival recession defects affecting the maxillary left anterior region. Root surface exposure is evident on teeth #9, #10, #11, and #12 (arrows), resulting in compromised gingival esthetics and increased susceptibility to dentinal hypersensitivity. The patient presented with concerns regarding root exposure and the appearance of the affected gingival tissues prior to treatment with the Zucchelli modification of the coronally advanced flap technique.

Clinical examination revealed multiple adjacent gingival recession defects involving teeth #9, #10, #11, and #12, with associated root exposure and esthetic concerns (Figure 1).

Periodontal evaluation demonstrated adequate plaque control, absence of active periodontal disease, and sufficient interdental soft tissue support. Based on the clinical findings, treatment with the Zucchelli modification of the CAF without connective tissue grafting was planned. Written informed consent was obtained prior to treatment.

**Figure 2:**
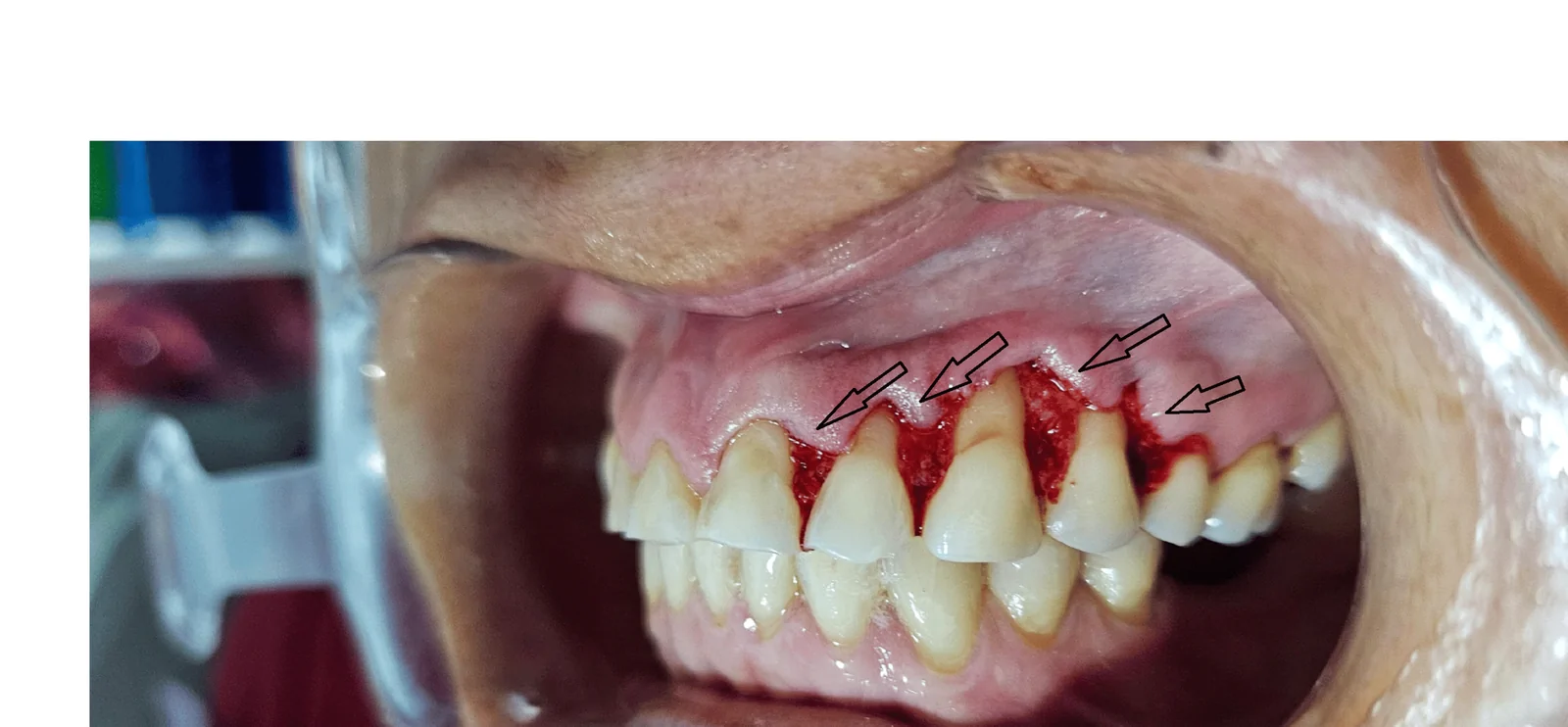
Zucchelli Modification of the Coronally Advanced Flap Design. Intraoperative clinical photograph demonstrating the Zucchelli modification of the coronally advanced flap following intrasulcular and oblique incisions. The surgical design was performed to treat multiple adjacent gingival recessions affecting teeth #9 through #12. The interdental papillae were preserved, and no vertical releasing incisions were utilized. The exposed recipient bed and flap design (arrows) illustrate preparation for tension-free coronal advancement and subsequent root coverage.

After administration of local anesthesia with 2% lidocaine containing 1:100,000 epinephrine, intrasulcular and oblique incisions were performed according to the Zucchelli modification of the CAF technique. The surgical design extended from the distal aspect of tooth #9 to the distal aspect of tooth #12, preserving the interdental papillae and avoiding vertical releasing incisions. A full-thickness flap was elevated in the coronal portion and continued as a partial-thickness flap apically to allow tension-free coronal advancement (Figure 2).

**Figure 3:**
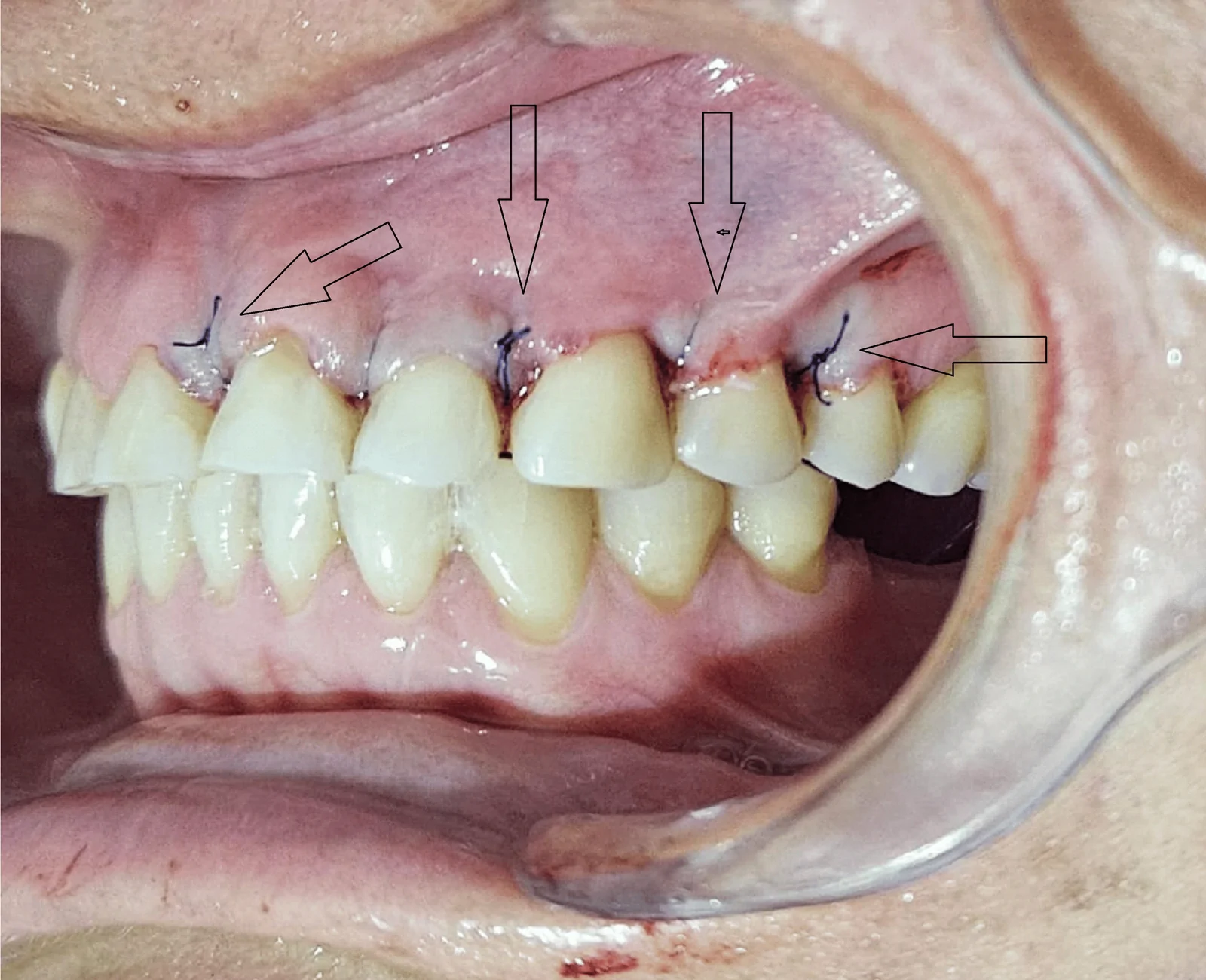
Early Postoperative Outcome Following the Zucchelli Modification of the Coronally Advanced Flap. Postoperative clinical photograph demonstrating successful coronal advancement and stabilization of the coronally advanced flap following treatment of multiple adjacent gingival recessions. Resorbable sutures (arrows) maintained flap adaptation and primary wound closure during the initial healing phase. Clinical evaluation revealed substantial coverage of the previously exposed root surfaces, improved gingival architecture, and favorable soft tissue integration. These findings demonstrate favorable clinical root coverage and esthetic improvement following the Zucchelli modification of the coronally advanced flap.

Following flap elevation, the exposed root surfaces were thoroughly debrided and prepared to receive the advanced flap. The flap was then repositioned coronally to completely cover the recession defects and stabilized using resorbable sutures (Figure 3).

Primary wound closure was achieved without tension, and postoperative instructions were provided.

**Figure 4:**
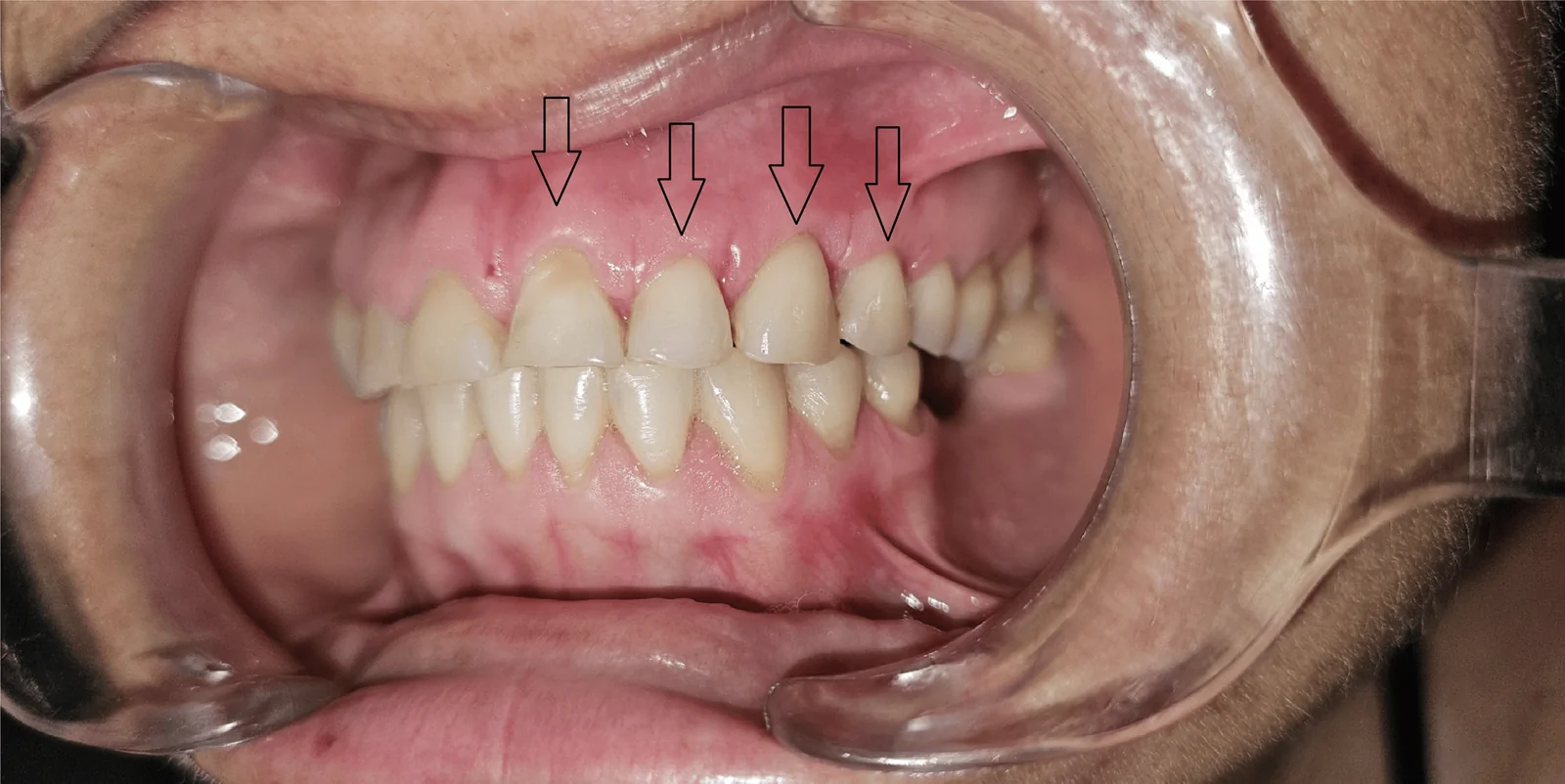
Postoperative Root Coverage Following the Zucchelli Modification of the Coronally Advanced Flap. Postoperative clinical photograph demonstrating successful healing and favorable root coverage following treatment of multiple adjacent gingival recessions using the Zucchelli modification of the coronally advanced flap. Clinical evaluation revealed improved gingival contour, increased soft tissue coverage over previously exposed root surfaces, and harmonious integration of the gingival margins. Arrows indicate the treated sites, where substantial root coverage and enhanced esthetic outcomes were achieved during the postoperative healing period.

Healing progressed uneventfully, with no postoperative complications or signs of infection. Follow-up clinical evaluation demonstrated favorable soft tissue healing, improved gingival contour, and substantial root coverage of the previously exposed root surfaces (Figure 4).

The patient reported significant improvement in both esthetics and reduction of root sensitivity and expressed satisfaction with the treatment outcome.

## Discussion

Gingival recession is a common mucogingival condition that may result in root exposure, dentinal hypersensitivity, plaque accumulation, root caries, and esthetic concerns [1]. Recent epidemiologic evidence confirms that gingival recession remains highly prevalent and is associated with multiple risk factors [2]. Periodontal plastic surgery has evolved considerably over the past decades and has become a predictable treatment modality for the management of recession defects [3,4].

Among the available root coverage procedures, the CAF has demonstrated favorable clinical outcomes and high patient satisfaction [5]. Zucchelli and De Sanctis reported favorable root coverage and esthetic outcomes using a modified CAF for the treatment of multiple recession type defects, supporting the use of this flap design for adjacent recession defects [6]. A subsequent systematic review and meta-analysis by Bhatia et al. further supported the efficacy of the modified CAF for the treatment of multiple adjacent gingival recessions [7]. Zucchelli et al. also demonstrated favorable clinical outcomes when CAF procedures were combined with connective tissue grafting, highlighting the potential contribution of grafting to soft tissue augmentation and root coverage [8,9]. More recent modifications have expanded the application of CAF techniques in regenerative procedures [10]. The Zucchelli approach for multiple recession defects in the maxillary anterior region remains an important reference in contemporary periodontal plastic surgery [11], while comparative randomized clinical studies have further supported the clinical effectiveness of different CAF designs [12].

Despite the predictability of root coverage procedures, complications and technical errors may influence treatment outcomes and should be carefully considered during treatment planning [13]. Contemporary reviews continue to support periodontal plastic surgery as an effective treatment modality for gingival recessions affecting both single and multiple teeth [14].

HIV infection has historically been associated with a variety of oral manifestations [15]. Periodontal plastic surgery has been extensively studied and refined over time [16-18]. In addition, current evidence highlights the importance of gingival phenotype and tissue thickness in determining the long-term stability of root coverage procedures [19,20].

The decision to perform the CAF without connective tissue grafting was based on the clinical objective of treating multiple adjacent recession defects while avoiding the additional donor-site morbidity associated with graft harvesting. Although connective tissue grafting may provide additional soft tissue thickness and favorable root coverage outcomes, randomized clinical evidence has also demonstrated favorable results with CAF-based approaches. In the present case, omission of the connective tissue graft eliminated the need for a second surgical site while allowing coronal advancement of the flap over the affected teeth. The favorable healing and root coverage observed clinically support the feasibility of this approach in this individual patient; however, the absence of standardized quantitative periodontal measurements and long-term follow-up limits direct comparison with graft-assisted procedures.

The patient’s medically controlled HIV infection was considered during case selection and surgical planning. Her adherence to antiretroviral therapy and absence of reported opportunistic infections were reviewed before treatment, and standard infection control and surgical aseptic protocols were followed. Postoperative healing was uneventful, with no reported infection or other complications. However, because specific CD4 count and viral load values were unavailable for verification, the influence of HIV status on the treatment outcome cannot be independently assessed. Therefore, this case demonstrates the feasibility of the Zucchelli modification of the CAF without connective tissue grafting in this carefully selected, medically controlled patient, rather than establishing the broader predictability of the procedure in patients with HIV infection.

## Conclusions

The Zucchelli modification of the CAF provided successful treatment of multiple adjacent gingival recessions, resulting in favorable root coverage, improved gingival contour, and enhanced esthetic outcomes. In this case, satisfactory healing and soft tissue stability were achieved without the use of a connective tissue graft. Furthermore, the medically controlled HIV positive patient experienced uneventful postoperative healing and a favorable clinical outcome. This case demonstrates that the Zucchelli modification of the CAF without connective tissue grafting was feasible in this carefully selected patient with medically controlled HIV infection. However, the absence of quantitative periodontal measurements and long-term follow-up limits broader conclusions regarding the effectiveness of this approach.
